# A Hospital for Soldiers Suffering from Shock

**Published:** 1914-12-12

**Authors:** Edwin Ash


### A HOSPITAL FOR SOLDIERS SUFFERING FROM SHOCK.

### Dr. Edwin Ash's Reply to Lord Knutsford.

Sir,—I have read Lord Knutsford's comments on my letter in The Hospital of November 28, and regret his personal tone, albeit his personalities are not so marked as in his *Observer* communication. Let us consider further a few of his points:

### (1) "We are just doing Different Work."

"The accommodation provided by the existing hospitals was not what was needed for a great number of these poor fellows . . ." For some reason or other, Lord Knutsford seems to think that in his hospital a new kind of disease engendered by the war is to be treated. But it is clear to everyone who has any knowledge of the subject that the traumatic neuroses of war do not differ essentially from the traumatic neuroses of civilian life, and neurasthenia due to shock has been treated with success by the existing hospitals for years upon years. If they can treat shock due to railway accidents, why cannot they treat nerve shock due to the stress of war? Does Lord Knutsford realise that his contention that the nerve hospitals in Queen Square, Maida Vale, and Welbeck Street are unfitted to care for soldiers sent home suffering from nerve shock means that these institutions are equally unfitted to deal with neurasthenia and allied conditions affecting the civilian population? Are we to understand that these institutions are not now, nor ever have been, capable of dealing in their wards with this extremely prevalent nervous disorder?

### (2) "Cubicle Accommodation is Useless."

That is a matter of opinion. And if Lord Knutsford cares to take a census of medical men having special experience of nervous diseases he will find that by no means all of them agree with him on this point. On the Continent, Professor Dejerine—whose name is honoured by neurologists in this country—is a firm believer in the efficacy of cubicle accommodation in the treatment of these cases. If statements in support of this coming from myself are "distilled rubbish"—whatever that may be—then I take it that the same dignified expression applies to them when made by Professor Dejerine or eminent neurologists in this country who happen to agree with his views. Apropos of this, I have been twice asked lately if Lord Knutsford is a doctor with special experience in nervous diseases? I leave him to answer the question.

Before going further, I may bring forward a little evidence that I am not alone in my views, and will quote from an unsolicited letter received from a well-known neurologist—a member of the staff of one of the special hospitals in question. Referring to my letter in the *Observer* (November 30), he says: "I regret that owing to my being employed in the Navy, I am precluded from in any way taking part in the controversy, but I wish it could be possible to point out to Lord Knutsford and the public in general that traumatic neurasthenia and allied neuroses and psychoses are not new diseases begotten of the war, but that they have been treated by the existing hospitals with success for as long as they have been founded. It should also be borne in mind that Professor Dejerine, an eminent exponent of the claims of isolation and psychotherapy, treats similar cases screened from each other in large wards. I entirely agree with you that the resources of existing hospitals should be made use of."

### (3) The "Signatories."

Lord Knutsford makes great play with the names of his "signatories." There were originally twenty-three names published as those of gentlemen agreeing with the scheme for a new nerve home. But it did not appear from Lord Knutsford's original letter that they had actually signed his appeal. These included those of twenty-two medical men and one well-known authority on psychological problems, who is not, I believe, a medical man. The list appended to his subsequent letters contains now twenty-one names of doctors and that of the aforementioned psychologist; evidently one of his followers has already dropped out. Do these gentlemen without exception actually consider themselves "signatories" to the original appeal or to subsequent statements in support of which their names have been freely posted in the public Press? There are many eminent neurologists whose names should also appear if the list is to be considered representative of current professional opinion.

### (4) The Staff of the New Hospital.

It is to be inferred that the staff of the new nerve home is to consist of twenty-two doctors already referred to, supported by the eminent psychologist. One wonders, then, why for a home which is to accommodate thirty or forty patients a staff of over twenty medical men is required. A great point has been made of the fact that even if the existing hospitals for nervous diseases can deal with these patients only the neurologists constituting their staffs could carry out the treatment. And why not? What is the objection to this? Why is it so necessary to place the soldiers suffering from nerve shock in charge of a certain group of medical men? What is the objection to the treatment being supervised by another group—to wit, those neurologists who form the staffs of the special hospitals who have not associated themselves with the new scheme?

### (5) "Rest is not all that is Needed."

"If it establishes the usefulness of strict isolation or of any special treatment for these nerve-shattered men its ephemeral existence will not have been in vain, and it will have done good work apart from the number of patients cured. . . .'' This suggests an experimental point of view. But this is not the time nor place for experimental therapeutics. After being so dogmatic as to the necessity for absolute isolation Lord Knutsford now raises the question of its importance, and tells us that the new hospital may possibly establish its usefulness! No further comment is needed here. Lord Knutsford and his staff owe it to the public and the medical profession to make clear just what it is that they expect to be able to do in the new home that they cannot carry out elsewhere. What are the special treatments that are to be used? Is there any reason why we should not be informed clearly what are to be the practical workings of the new institution in regard to treatment?

In conclusion, I may point out that I am fully in agreement with funds being raised to assist the class of sufferer he has in view, although I think that the institution of a new special hospital at the outset is a wrong move. However, ultimately the publication of its statistics will probably prove my point that the existing nerve hospitals can do just as well in the treatment of traumatic neurasthenia and allied conditions.

